# Cross-platform mass spectrometry annotation in breathomics of oesophageal-gastric cancer

**DOI:** 10.1038/s41598-018-22890-w

**Published:** 2018-03-23

**Authors:** Sung-Tong Chin, Andrea Romano, Sophie L. F. Doran, George B. Hanna

**Affiliations:** Department of Surgery and Cancer, Division of Surgery, Imperial College London, London, W2 1NY United Kingdom

## Abstract

Disease breathomics is gaining importance nowadays due to its usefulness as non-invasive early cancer detection. Mass spectrometry (MS) technique is often used for analysis of volatile organic compounds (VOCs) associated with cancer in the exhaled breath but a long-standing challenge is the uncertainty in mass peak annotation for potential volatile biomarkers. This work describes a cross-platform MS strategy employing selected-ion flow tube mass spectrometry (SIFT-MS), high resolution gas chromatography-mass spectrometry (GC-MS) retrofitted with electron ionisation (EI) and GC-MS retrofitted with positive chemical ionisation (PCI) as orthogonal analytical approaches in order to provide facile identification of the oxygenated VOCs from breath of cancer patients. In addition, water infusion was applied as novel efficient PCI reagent in breathomics analysis, depicting unique diagnostic ions M^+^ or [M-17]^+^ for VOC identification. Identity confirmation of breath VOCs was deduced using the proposed multi-platform workflow, which reveals variation in breath oxygenated VOC composition of oesophageal-gastric (OG) cancer patients with dominantly ketones, followed by aldehydes, alcohols, acids and phenols in decreasing order of relative abundance. Accurate VOC identification provided by cross-platform approach would be valuable for the refinement of diagnostic VOC models and the understanding of molecular drivers of VOC production.

## Introduction

Stomach and oesophageal cancers are the fifth and eighth most common cancers in the world with 952,000 and 456,000 new cases diagnosed in 2012^1^. In the early stages, OG cancer is often associated with non-specific symptoms commonly experienced by patients with benign disease. When ‘alarm’ symptoms do occur, it is often a sign of advanced disease with poor long-term survival. Whilst earlier diagnosis will improve long term survival, existing strategies for detecting these cancers are invasive and expensive making them unsuitable for use in the vast numbers of patients who present to medical services with upper gastrointestinal symptoms of uncertain origin. There is a need for a non-invasive test to triage patients with non-specific upper gastrointestinal symptoms to have endoscopy in attempt to rationalize patient diagnostic pathway. Such triage test needs to have specific characteristics: simple to administer, cheap, of appropriate accuracy and has high patient and staff acceptance rate. A breath test based on the detection of VOCs lands itself into this category.

Development of non-targeted breathomics analysis for disease diagnosis is of great interest, since it investigates VOCs in exhaled breath as indicators of the patient’s metabolic status^2,3^. Specific range of oxygenated VOCs detected from the exhaled breath have been reported as possible biomarkers in cancer diagnosis, distinguishing between malignant and non-malignant conditions as well as patients with different stages of precancerous gastric lesions^4–6^. VOC composition of breath is typically complex, comprising multitudes of metabolite compounds down to parts-per-trillion in concentration. Direct injection MS has been demonstrated as high-throughput breathomics tool in search of pattern recognition or composite biomarkers on disease phenotyping^7^. SIFT-MS offers the advantage of real-time quantification of several trace gases simultaneously in breath, obviating the need for sample breath concentration, derivatisation method and calibration^8^. Lately, Kumar *et al*.^9,10^ demonstrated the potential of exhaled breath VOC analysis using SIFT-MS to distinguish the patient groups with OG cancer from that of benign disease and healthy upper gastrointestinal tract cohorts. Nevertheless, the external validation of VOC diagnostic studies is very challenging with a paucity of reports on phase II independent multicenter studies in-spite of the presence of multiple phase I reports associating VOCs with specific cancer sites.

VOCs verification by SIFT-MS can only be conducted according to the kinetic database established through ion-molecule reaction with selected reagent/precursor ions. The verified VOC mass peak can be ambiguous especially for isobaric and isomeric molecules, thus validation of the distinguished biomarker identity with another orthogonal technique would be critical. Annotation of the distinctive VOC marker peaks is crucial since all substances involved have to be characterised unequivocally and the patho-physiological pathways have to be elucidated and understood. GC-EI-MS is regarded as the mainstream VOC analytical method which incorporates VOC separation prior to MS detection. This allows detailed VOC identification although this analysis requires mass spectral libraries, yet to reflect all VOCs^11^. This may result in pending conclusion on the chemical structure of detected VOCs. Integration of soft CI profiles in GC-MS analysis could prove valuable as it results in complementary information that aids the identification of unknown VOCs^12,13^. However, exploration of breath VOC by GC-CI-MS is scarcely reported.

In order to extract knowledge from breathomics datasets in the clinical analysis, an interactive analytical strategy across multiple MS-based platforms is extremely useful which offers a shared standard to achieve readily comparable global data consultation. This work aims to demonstrate a cross-platform analytical strategy in a complementary manner of two thermal desorption unit (TD) coupling MS platforms, namely TD-SIFT-MS and TD-GC-MS, described for oesophageal-gastric cancer breathomics under clinical environment. Analytical performance across the MS platforms was evaluated for profiling exhaled breath VOC focusing on the potential biomarker groups i.e. phenol, acid and aldehyde groups, reported previously by Kumar *et al*.^9,10^. Integrated TD-GC-MS with high energy EI and distinctive CI approaches was addressed for identification of exhaled breath VOC markers.

## Materials and Methods

### Chemicals and Reagents

Neat chemicals as VOC reference standards of over 97% purity as well as Chromasolv grade methanol were obtained from Sigma-Aldrich (St. Louis, USA). Ultrapure water was acquired from Direct-Q3UV water purification system (Merck KGaA, Darmstadt, Germany). Stock solutions of individual compounds were prepared by diluting standards to 1 g mL^−1^ with methanol and stored at −20 °C prior to analysis within 4 weeks.

### Preparation of VOC standards loading onto TD tube

VOCs were loaded onto TD tubes using two different methods, either liquid or gas injection for the purposes of qualitative and quantitative evaluation respectively. For liquid standard loading, 0.5 µL of VOC standard mixture as listed in Table S-1 at 100 ng µL^−1^ were injected into individual TD tubes using a GC syringe via a Markes Calibration Standard Loading Rig (CSLR). The GC syringe needle was kept within the loading rig for a short interval of 20 seconds to achieve complete evaporation of target analytes. Nitrogen flow was set at 50 mL min^−1^ to aid the retention of the VOC onto the sorbent beds and remove the methanol solvent from the tube. For vapour standing loading, a permeation unit and device model ES4050P (Eco Scientific, Gloucestershire UK) were employed. The permeation device consisted of a PTFE tubing section at 100 mm in length, filled with the compounds of interest, and kept under 30 °C at 1 L min^−1^ air flow in order to provide permeation-driven flow of VOC over time constantly. Concentration of analyte included phenol, butanal and butanoic acid were equilibrated at 1 × 10^−10^ mol, 4 × 10^−10^ mol and 15 × 10^−10^ mol respectively. Tube loading was achieved by connecting the outlet directly to the TD tube whilst the sampled amount was adjusted using different loading times for response linearity test.

### Breath sample collection

Exhaled breath from 21 patients was sampled onto TD tubes (pre-packed with 200 mg of Tenax and 100 mg of Carbograph5) purchased from Markes International (Llantrisant, UK). Noted that the recruited samples were intended to evaluate the compound annotation of VOCs via multiple MS platforms, higher power would be required to attempt statistical modeling. Ethical approval was obtained through NHS Health Research Authority (NRES Committee London – Camden and Islington) which gained on 16^th^ July 2014 (REC reference 14/LO/1136). Recruitment and all sampling methods were carried out at St Mary hospital, London in accordance with our previous published protocol^14^ using ReCIVA breath sampler (Owlstone, Cambridge, UK)^15^. The patients undergoing upper gastrointestinal endoscopy at Imperial College Healthcare NHS Trust provided written informed consent before breath sampling. Demographic and clinical information were collected (supplementary information Table S-2).

**Figure 1:**
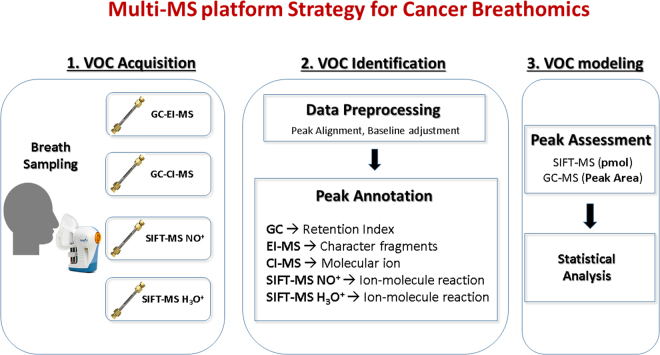
Multi-MS platform strategy for cancer breathomics involving analytical flow path from exhaled VOC sampling, mass peak identification, compound assessment and statistical analysis. List of molecular details was acquired by complementary MS platforms for enhanced confidence of VOC annotation.

Prior to sampling, tubes were conditioned at 325 °C for 40 minutes in a stream of nitrogen passed through a hydrocarbon trap (Supelco, US) using a Markes International TC-20 tube conditioner, operated according to manufacturer’s recommendation. Four TD tubes was used in ReCIVA to simultaneously collect the VOCs from a patient’s breath for later *in-vitro* analysis. Whole breath was collected according to our previous procedure^15^ but at 250 mL air volume using 400 mL min^−1^ flow rate. Once the collection was complete, the sorbent tubes were removed from the sampler device and stored under −80 °C with brass cap lock on both ends of the tube. All TD tubes were subjected to GC-MS and SIFT-MS analysis within 4 weeks of storage. A previous study indicated that the maximum storage duration under −80 °C condition on a dual bed TD tube was 1.5 month with 94% of the VOCs stable^16^. An activated charcoal purifier on inhaled air was retrofitted and room air was sampled in order to verify and minimize the exogenous factors in the analysis. Figure 1 illustrates the proposed multi-platform workflow encompassing breath VOC acquisition, peak annotation and assessment for cancer breathomics.

### TD-SIFT-MS operating condition

Captured VOCs were released by a thermal desorption instrument (TurboMatrix 300 TD, PerkinElmer, USA) and analyzed by a Profile-3 SIFT-MS instrument (Instrument Science, Crewe, UK)^17,18^. Primary desorption was performed by pre-purging the TD tube for 1 min with helium at 50 ml min^−1^ and heated to 280 °C for 10 min to desorb the VOCs onto a cold trap containing Tenax GR backed by Carbopack B. During secondary desorption stage, the cold trap was initially cooled to 10 °C and heated to 290 °C at 5 °C sec^−1^ and hold for 4 min. The instrument had a head pressure of helium set to 8.5 psi which resulted in a flowrate of 100 mL/min within the transfer line between the TD device and the SIFT-MS at splitless mode. Ionisation was carried out using either H_3_O^+^ (m/z 19, 37, 55 and 73) or NO^+^ (m/z 30, 48 and 66) as precursors with a total count rate of approximately 8 × 10^5^ or 4 × 10^5^ counts s^−1^, respectively. Multi-ion monitoring mode (MIM) was mainly employed for this study. Desorbed VOCs were analysed for a total of 60 s, and the measured concentrations were averaged over the analysis time for each VOC.

### TD-GC-MS operating condition

An Agilent 7890B GC with 5977 A MSD (Agilent Technologies, Cheshire, UK), coupling to a Markes TD-100 device was used. Details for TD and GC-EI-MS operation was programmed according to previous work^15^. Briefly, the TD tube sample was pre-purged for 1 min at 50 mL min^−1^ constant helium flow rate prior to 280 °C for 10 min. This was followed by secondary desorption which heated the cold trap (U-T12ME-2S; for materials emissions C4 to C32) rapidly from 10 °C to 290 °C at 99 °C/min heating rate and held for 4 min to completely transfer the VOCs onto GC. Flow path from TDU to GC was heated constantly at 140 °C. VOC separation was performed on a ZB-624 capillary column (60 m × 0.25 mm ID × 1.40 µm *d*_*f*_; Phenomenex Inc, Torrance, USA) programmed at 1.0 mL min^−1^ Helium carrier. Oven temperature profile was set at 40 °C initially for 4 min, ramp to 100 °C (5 °C min^−1^ with 1 min hold), ramp to 110 °C (5 °C min^−1^ with 1 min hold), ramp to 200 °C (5 °C min^−1^ with 1 min hold), final ramp to 240 °C at 10 °C min^−1^ with 4 min hold. The MS transfer line was maintained at 240 °C whilst 70 eV electron impact at 230 °C was set while the quadrupole was held at 150 °C. MS analyzer was set to acquire over the range of 20 to 250 m/z with data acquisition approximated to 6 scan sec^−1^. For PCI-MS analysis, a standard 5975 CI manifold was used. Briefly, liquid reagent was contained in a standard 100 mL HPLC bottle, whilst the bottle headspace was introduced into the CI manifold via a Teflon tubing (1 m length × 0.317 cm o.d.) with a 1/8-inch Swagelok connector. The reagent gas flow were conducted at 15% of the total flow of 5 mL min^−1^. PCI autotune with methane reagent gas was used for the initial source tuning parameters and lens voltages. Mass spectra were recorded in full scan ranging from 50 to 400 m/z.

### Data Processing

GC-MS data was processed using MassHunter software version B.07 SP1 (Agilent Technologies) while MS data of the separated VOC component was compared with NIST Mass Spectral Library (National Institute of Standards and Technology version 2.0) for compound identification. All statistical analysis was performed using IBM SPSS 24 (IBM corp., Armonk, NY) to identify significant differences between the demographic factors as listed in Table S-2.

## Results

### GC analysis of VOCs

Table S-1 lists out the molecular details of VOCs verified using GC-EI-MS, GC-PCI-MS and SIFT-MS based on authentic standard mixture comprising multiple chemical classes of phenol, acid, ketone and aldehyde groups. By introducing saturated alkane mix ranging from C5 to C20 through TD tube onto GC column, specific retention index (RI) for individual VOCs was generated on this column phase and used as benchmark to monitor GC analysis throughout the study. Resolution of individual VOCs from a complex breath matrix is critical, because well resolved chromatographic peaks result into high quality mass spectra definition, thus enhancing identification capability. The mid-polar 624-phase capillary column with thicker film coating was efficient for GC resolution of highly volatile components such as propanal (RI 489) from isobaric acetone compound (RI 498) with boiling point as low as 46 °C. Compared to the MS techniques^9,10^, GC analysis shows its privilege by efficiently distinguishing ethylphenol isomers in the VOC mixture. In particular, 2-ethylphenol (RI 1286) eluted earlier than 3-ethylphenol and 4-ethylphenol although the latter two were overlapped (RI 1323). Such compounds exhibit similar mass spectrum as well as product ion generated by all different precursor ions in SIFT-MS analysis. In order to achieve efficient isomeric separation on methylphenol, polyethylene glycol or Wax phase with higher hydroxyl affinity properties was suggested in the NIOSH standard method^19^.

### EI-MS identification of VOCs

**Figure 2:**
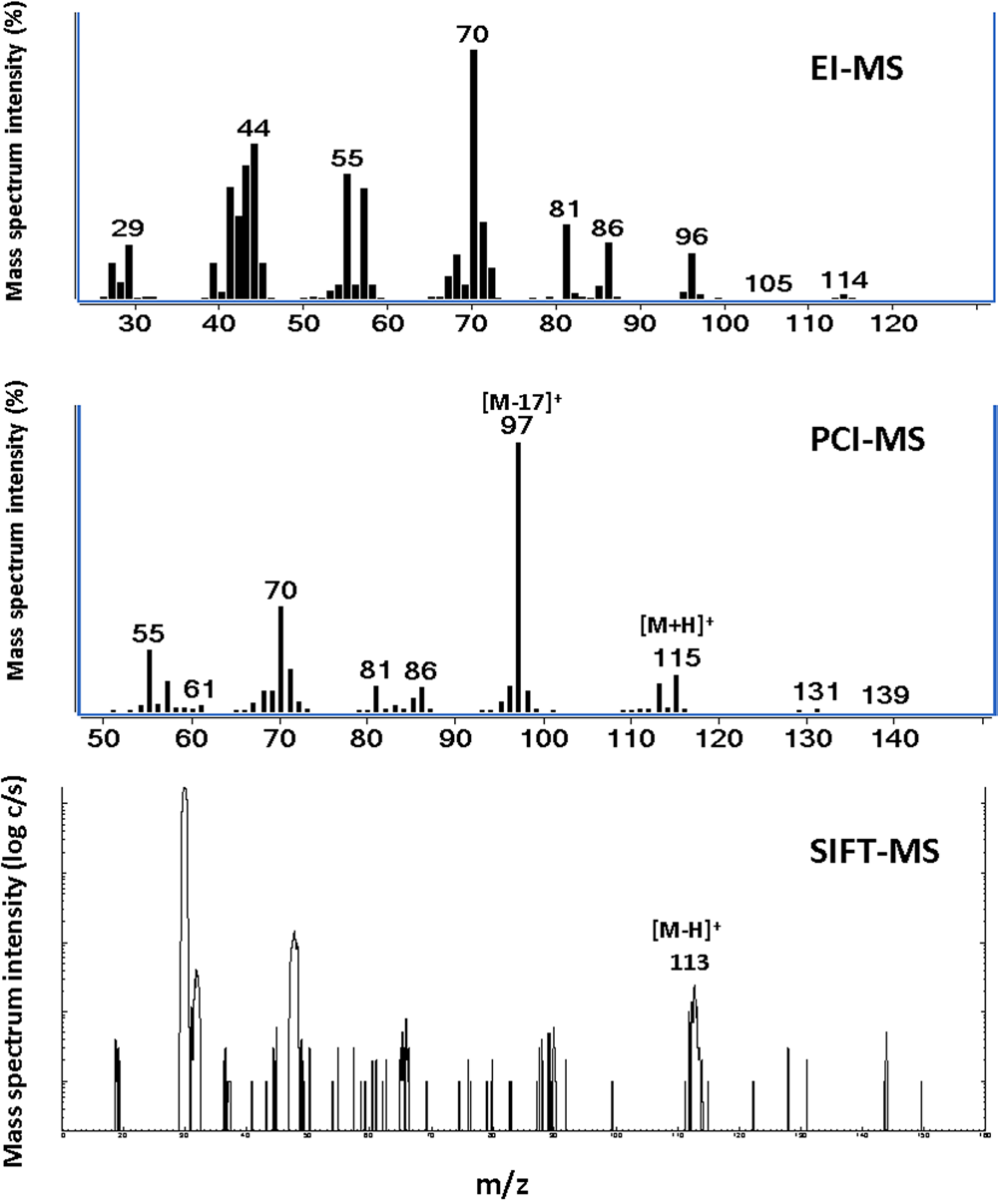
Mass spectrum on heptanal as representative VOC acquired by different MS platform (**A**) EI-MS, (**B**) PCI-MS, (**C**) SIFT-MS scan mode. Significant mass fragments in each MS systems were indicated as character ions for identification of compound molecule.

In general, aliphatic VOC ketones underwent α-cleavage fragmentation in EI-MS (Table S-1) which resulted in abundant [C_2_H_3_O]^+^ fragment at m/z 43 but low-intensity molecular ions (M^+^). Likewise, linear saturated aldehyde afforded spectra with absence of M^+^ but abundant m/z 43 and m/z 44 ions were observed, which were likely due to α-cleavage fragmentation and McLafferty rearrangement respectively. Figure 2 displays the comparison of mass spectrum of heptanal acquired by EI-MS, PCI-MS and SIFT-MS. Meanwhile, unsaturated secondary aldehydes displayed [H_2_C‒C≡O]^+^ fragment of m/z 41 dominantly as the product of β-cleavage fragmentation. Such phenomenon does not occur for smaller C3 aldehyde molecules, i.e. propanal and propenal, which preserve their M^+^ with moderate HC=O dissociation to produce [M-29]^+^ fragment.

Shorter carbon-chain acid VOCs such as acetic acid and propanoic acid could be distinguished by prevalent [M-17]^+^ peaks whilst longer chain carboxylic acids were better identified by the fragments at [C_n_H_2n-1_O_2_]^+^ and the McLafferty peak at m/z 60 which accounted for the base peak. Linear primary alcohols VOC could be identified via the characteristic peak at [M-H_2_O]^+^, [M-33]^+^, and [H_2_COH]^+^ of m/z 31. Identification of the alcohol molecule was complicated by the prevalence [M-H]^+^ due to loss of hydride ion from the α carbon. Lastly, aromatic VOCs including phenol, methylphenol, and benzaldehyde displayed their distinct M^+^ peak, as shown in Table S-1. A distinctive [C_6_H_5_]^+^ peak at m/z 77 in the spectrum of methylphenol, ethylphenol and benzaldehyde was indicative of the substituted benzene ring. As the mentioned ion fragments were highly similar among each chemical family, difficulty arise in identification when matching of unknown VOC with MS databases is unambiguous.

### PCI-MS identification of VOCs

Table S-1 lists out the abundant mass ions generated by water reagent PCI amongst the tested VOC mixture. Preliminary test on CI reagent revealed that application of water infusion (proton affinity, PA = 697 KJ mol^−1^) exhibited higher efficient in promoting ionisation of the oxygenated VOC mixture than that of commonly used methane CI reagents (PA = 552 KJ mol^−1^). Water infusion at 40 eV induced H_3_O^+^ ion which functions as a Bronsted acid while the energy transferred to the sample molecule under protonation reaction is relatively minor and the resulting [M + H]^+^ ions are stable toward fragmentation. As indicated in Table S-1, [M + H]^+^ were typically observed as the base peak for phenol, ketone, unsaturated aldehyde and acid group. Longer chain saturated aldehydes (>C4) constituted a minor [M + H]^+^ ion with relative intensity of < 30% in PCI-MS analysis (Table S-1). Instead, [M-17]^+^ and [M-73]^+^ exhibited the most intense peak in these saturated aldehyde which correspond to dissociation of OH and inductive cleavage fragmentation (Fig. 2). Meanwhile, alcohols has undergone hydride abstraction resulting intense [M-H]^+^ peak whilst larger alcohols (>C3) experienced loss of OH to generate [M-17]^+^ base ion.

### SIFT-MS identification of VOCs

Coupling of TD to SIFT-MS requires careful consideration on adjusting flowrate of inlet gas in SIFT-MS for accurate ion quantification. The TD desorption was programmed to achieve 30 mL min^−1^ in order to be compatible with the flow tube working conditions, resulting in a base peak width ranging from 20 to 30 s with water level below 500 ppm. SIFT-MS measurement of selected VOCs is relied upon the employment of an up-to-date kinetic library established at MIM which is listed in the supplementary information (Table S-3). Library entries and the methods for their creation are reported in the literature for phenols^20^, ketones^21^, aldehydes^22,23^, alcohols^24^ and acids^25^. Overall, SIFT-MS displays a prevalent generation of protonated, molecular or quasi-molecular ions and respective adducts (Table S-1). Fragmentation is relatively low but this is also depending on the VOC and precursor ions considered. When using H_3_O^+^ precursor, the main reaction channels are protonation and water adduct formation for phenols, alcohols and acids, whereas dehydration also occurs for alcohols. NO^+^ percursor is particularly versatile, with different reaction channels accessed by the various chemical classes: phenols react undergoing electron transfer or water adduct formation; other reaction channels are nitrosonium adduct formation (ketones, unsaturated aldehydes, acids) and hydride abstraction (aldehydes). Among all examined classes of compounds, only acids undergo dissociative ionisation with NO^+^.

### Inter-comparison of MS platforms for VOCs assessment

**Figure 3:**
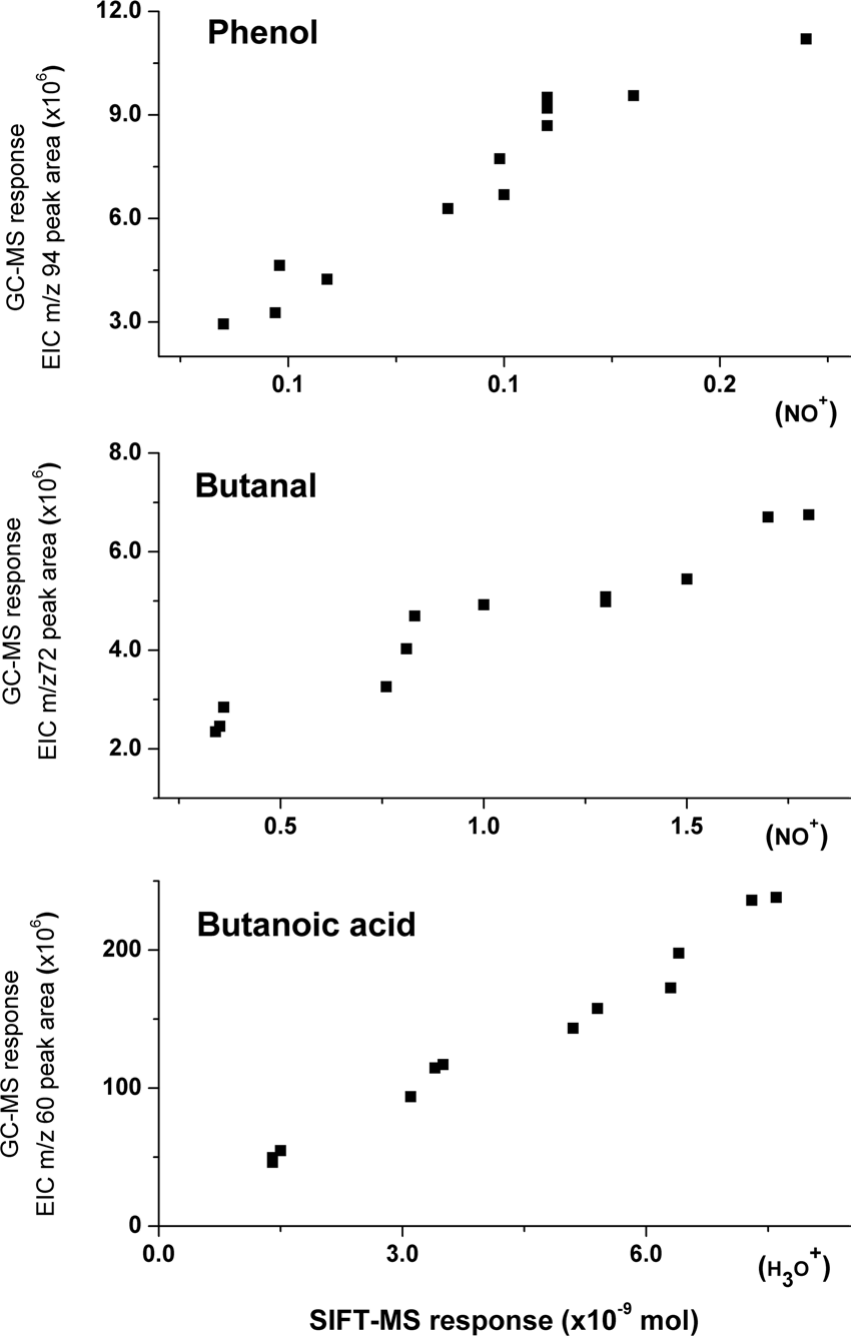
Intercomparison between SIFT-MS and GC-EI-MS responses on measurement of representative VOCs comprising phenol, butanal and butanoic acid. Vapour volume ranging from 50 mL to 1000 mL sampled from gas phase permeation. SIFT-MS operation was performed using suitable precursor ion namely NO^+^ and H_3_O^+^ for better selectivity on the tested VOCs.

Since limited number of compounds can be prepared with the permeation device, three VOCs were selected which have been previously reported as the potential biomarkers in oesophago-gastric cancer^9^. This is justified based on from different chemical groups namely aldehyde, acid and alcohol as well as application of different reagent ions i.e. H3O+ and NO+ in SIFT-MS analysis in order to achieve the best and most specific response on VOCs assessment. Inter-comparison between SIFT-MS and GC-EI-MS response measurements of butanal, butanoic acid and phenol was shown in Fig. 3. Paired TD tubes loaded with the aforementioned components concurrently with different time length from the permeation source were subsequently subjected to SIFT-MS and GC-EI-MS analysis. PCI-MS did not suit for absolute quantification of VOCs as PCI-MS generally exhibited lower sensitivity (approximately by 3-fold in average) than EI-MS which was also indicated in previous work^26^. Table 1 depicts the system performance on the representative VOCs quantitatively by comparing the detector responses between GC-EI-MS and SIFT-MS analysis. Correlation of detector response between the two platforms appears to be linearly fitted with correlation coefficient (R^2^) at 0.97, 0.94 and 0.92 for butanoic acid, butanal and phenol respectively. Quantitative VOC measurement of TD-SIFT-MS at both H_3_O^+^ and NO^+^ precursor satisfactorily agrees with TD-GC-EI-MS. Unlike GC-MS which requires calibration plot for assessment of each VOC, SIFT-MS allows target VOC quantitation without pre-separation. The relative standard deviations (n = 3) of the SIFT-MS method were 9% for both butanal and butyric acid but 22% for phenol. Limits of detection for the tested VOCs by SIFT-MS were estimated ranging from 2 to 7 ppbv regardless of the percursor type (Table 1). Once annotation of the significant compound has been verified by cross-platform analysis, SIFT-MS would become a useful option to conveniently quantify and monitor the changes of such compound without the need of calibration construction.

**Table 1:** Intercomparison between GC-EI-MS and SIFT-MS responses, SIFT-MS performance on the representative VOCs.

	Butanal	Butanoic acid	Phenol
Correlation equation of Intercomparison	y = 3E^ + 15 ^x+ 2E^ + 06^	y = 3E^ + 16^x + 7^E + 06^	y = 7E^ + 16^x + 792839
Correlation coefficient of Intercomparison	0.938	0.9792	0.9262
SIFT-MS relative standard deviation	9%	9%	22%
SIFT-MS limit of detection (ppbv)	4	2	6

Intercomparison between GC-EI-MS and SIFT-MS responses on the representative VOCs namely phenol, butanal and butanoic acid, with SIFT-MS quantitative performance indicating comparative method sensitivity and reproducibility.

### VOC analysis in cancer breath

**Figure 4:**
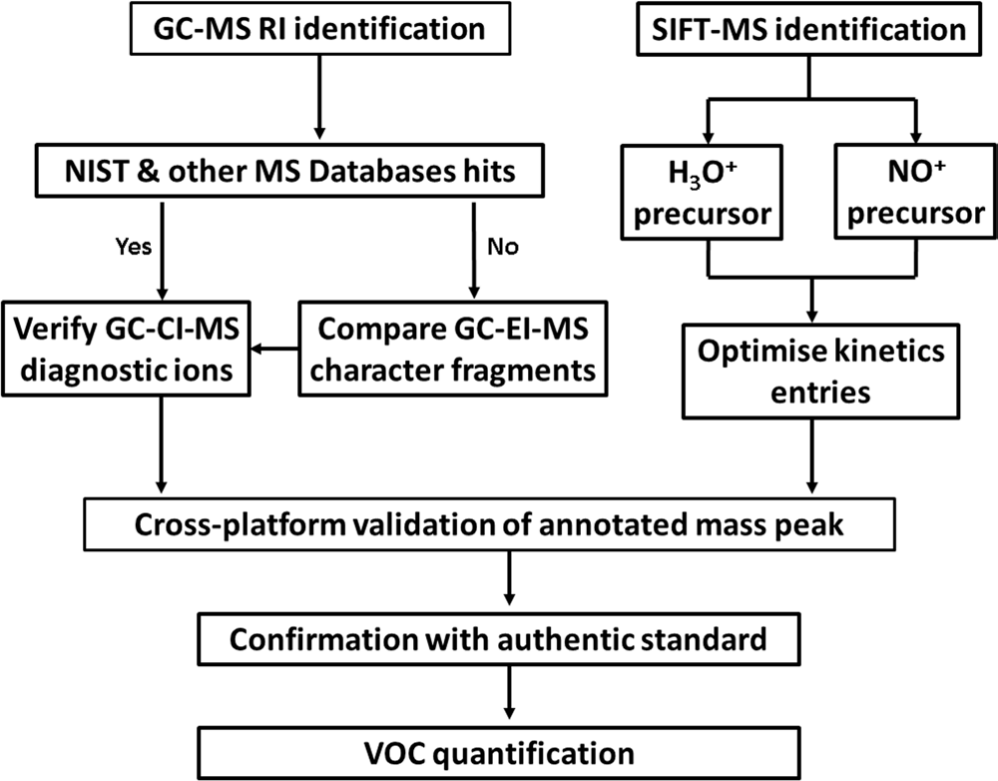
Cross-platform analytical workflow encompassed GC-PCI-MS and GC-EI-MS platforms as well as SIFT-MS with NO^+^ and H_3_O^+^ precursors on mass peak annotation of breath VOCs. Multiple MS platforms acquire complementary molecular details for validation of annotated mass peak of VOC.

Figure 4 illustrates the route map integrating RI, EI-, PCI- and SIFT-MS analysis for oxygenated VOC identification and discovery. The quadrupole mass spectrometer reliably assesses low molecular mass VOCs down to water mass at m/z 18. Verification of carbonyl VOCs is performed through *in situ* molecular ionisation and reaction within the instrumental chamber neglecting interference of artifacts prior to GC separation. Consequently, structure interpretation information obtained from the two orthogonal MS platforms can be combined and assessed for validity of mass peak annotation. Figure S-1 illustrates the representative total ion chromatograms obtained using GC-EI-MS and GC-PCI-MS as well as SIFT-MS on breath VOCs sampled from OG cancer patient. Noted that retention time of the analyte was neglected between the two GC-EI-MS and GC-PCI-MS analyses. Number of peaks was relatively lower in GC-PCI-MS analysis in which less polar VOCs such as siloxane (RT 11.89 min) exhibit less tendency to be ionised by the protonated water. Trace levels of dodecane (RT 38.11 min) were yielded by PCI-MS, which is in agreement with previous work^12^, whereas VOCs containing unsaturated carbon bonds, such as isoprene and limonene, were distinctly identified in the breath.

The effectiveness of the multi-platform strategy in providing more robust peak annotation was evident in the case of some oxygenated VOCs. For instance, for the chromatographic peak at RT 8.902 min in the total ion chromatogram (Figure S-1), low MS library hit was obtained (match and reverse match scores below 800), which did not permit robust peak annotation. Following the predictive process, m/z 29 fragment and m/z 59 protonated ion were distinguished in EI-MS and PCI-MS respectively for the aforementioned peak. In agreement with SIFT-MS detection via NO^+^ percursor reaction, propanal has fulfilled the criteria and hence been identified. Likewise, annotation confidence of acetic acid (RT 15.59 min) was improved according to the workflow, indicating abundant m/z 60 and 43 character fragments at EI-MS, m/z 61 or [M + H] ion at PCI-MS, as well as product ions m/z 61, 79 and 97 in SIFT-MS via H_3_O^+^ percursor. The deconvoluted MS spectrum of such peak in EI-MS was commonly below 800 of NIST library hit which induced misidentification with other candidates such as ammonium acetate. Analytical data obtained by PCI-MS provided mass spectral fingerprints that did not correspond with the results expected for ammonium acetate (MW 77 Da), thus screening out this possibility.

**Figure 5:**
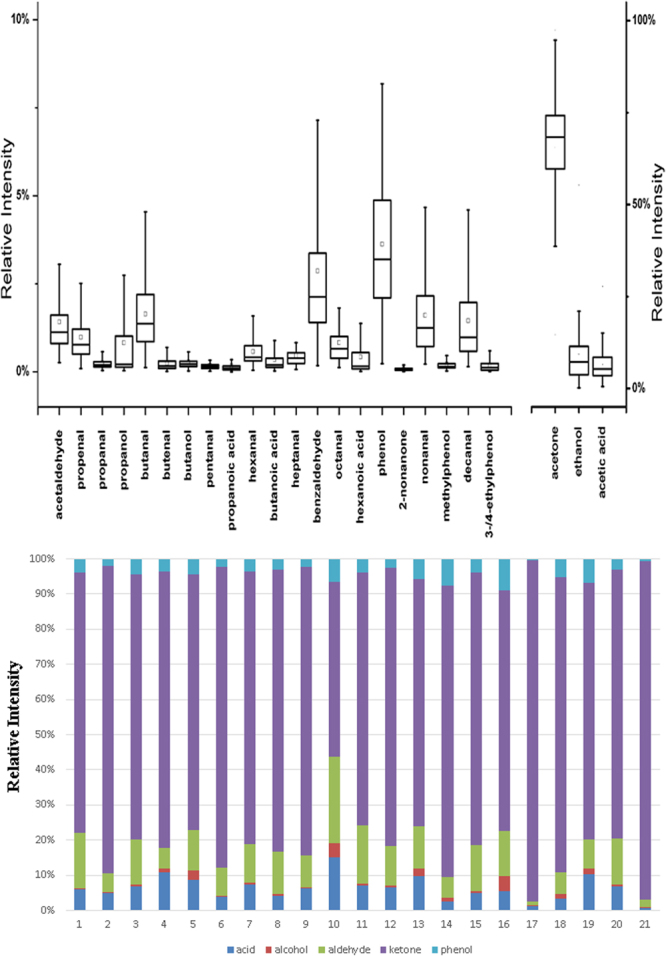
(Top) Box & Whisker plot of the relative intensity of VOCs composition in the exhaled breath of the OG cancer groups analysed by GC-EI-MS. (Bottom) Variation of the exhaled breath comprising different oxygenated VOCs groups among the recruited OG cancer patients. Relative intensity was derived from total peak area of identified oxygenated VOCs in exhaled breath.

The cross-platform workflow presented herein enables high confidence mass peak annotation and subsequently reveals the composition of oxygenated VOCs in exhaled breath of cancer patients. Distribution of the identified oxygenated VOCs from breath and their inter-variability among the recruited OG cancer patients was illustrated in Fig. 5. GC-PCI-MS is the least sensitive among the three MS platform (5 to 10-fold than EI-MS) whilst SIFT-MS is unable to distinguish branched chain alcohol and isomeric ethyl phenol. Thus, it is more sensible to use GC-EI-MS for profiling of the cancer breath VOCs justified from its broad molecular coverage, high resolution and detection sensitivity properties. Amongst the identified oxygenated components, ketones were the most represented with median 77% relative abundance, followed by aldehydes at 10%, acids at 9%, phenols at 3% and lastly alcohols at 22%. Kumar *et al*.^9^ depicts 13 VOCs comprising fatty acid, aldehyde and phenolic groups at significantly higher concentrations in both gastric and esophageal adenocarcinoma cohorts^9^. The chi-square test indicates that there is no statistically significant relationship (p > 0.05) between the VOCs variables and demographic factors. Meanwhile, independent t-test shows significant mean difference (p < 0.05) on diabetes factor amongst the acid, aldehyde and ketone variables. In particular, acid and aldehyde were elevated but ketone decreased amongst the patients diagnosed with diabetes. It also shows that acid increased significantly while ketone was significantly lower on patients with hypertension. Oxidative stress and related metabolic abnormalities occur in patients with diabetes and hypertension were likely contributed to the elevated level of acid and aldehyde^27^.

Acetone affords the highest contribution as shown in Fig. 5. The applicability of breath acetone as biomarker in clinical practice has been proposed as an indicator of ketosis related to alcoholism, or inborn error of metabolism^28^. Nevertheless, its concentration can also be influenced remarkedly by other non-clinical parameter such as diet and exercise since there is number of metabolic pathways such as citric acid cycle and Krebs cycle involve production of acetone. Thus, its suitability as cancer marker requires further verification. Ethanol and acetic acid constitute a relatively high proportion of the VOC profile with 7% and 5% relative abundance, respectively. Volatile aldehyde homologues, which are also known as one of the lipid peroxidation-derived products, were present as second dominant oxygenated VOCs in the exhaled breath of cancer patients. Notably, significant amount of acrolein and benzaldehyde were confirmed in the OG cancer breath which did not reported previously in the breath of gastric cancer patients^4,9^. However, acrolein were increased in the headspace of surgical lung cancer tissue specimens as a product from spermine metabolism^29^ while both aldehydes were previously suggested as potential volatile marker in lung cancer detection^30–32^. The result has also discriminated the presence of 2-ethylphenol in the breath of OG patients which narrow down the investigation on the metabolic regulation to 3- and 4-ethylphenol.

## Discussion

Analysis of VOCs from breath is predominantly influenced by volatile sampling method. In the previous studies^14^, breath samples were collected in a bag before being subjected to direct injection SIFT-MS analysis. Despite the stability of VOCs within steel bags for 48 hours, breath sampling in storage bags exhibits transportation and stability challenges in routine clinical practice between clinical sites and the laboratory. This study employs TD tubes for breath sampling to overcome such limitation whilst 4 sampling tubes can be applied to acquire breath VOC simultaneously. Provided constant flow rate going through the tubes is maintained, the VOCs profile amongst the sampled tubes remain insignificant. Simultaneous acquisition of 4 tubes minimizes the intra-sample variation and reduces the burden to cancer patient on repeated sampling procedure. Reduced amount of VOC collection occurs due to splitting of breath volume which could be compromised by adequately adjusting the sampling time and flowrate within the ReCIVA device. Nevertheless, discrimination on VOCs entrapment within the TD tube might be occurred due to affinity nature of the absorbent. For instance, butanone was found in the patients with gastric cancer using solid phase microextraction sampling^4^ but not detected in the breath profile here.

Identification of unknown VOCs in EI-MS is mainly based on the matching of mass spectrum generated by hard EI at 70 eV with that of the commercial available databases such as NIST or Fiehn library. For PCI-MS analysis of breath VOCs, other liquid reagent with greater PA such as methanol (PA at 761 KJ mol^−1^) generated distinctive amount of dimers and trimers that may interfere the interpretation of compound identity^33^. Contrary to a previous study^34^ showing that PCI of 4-decanone with water reagent also afforded excessive [2 M + H]^+^ of m/z 313, no dimer peak formation was observed in the ketone analysis performed in this work. Water infusion was previously reported in atmospheric pressure chemical ionisation analysis^35^ which favoured the detectability and reproducibility of metabolome derivatives.

Contrary to EI-MS, PCI-MS with water reagent allows relatively soft ionisation of VOC analytes under vacuum condition predominantly on proton transfer ionization mechanism, and hence generates significantly less fragmentation. The [M + H]^+^ or [M-17]^+^ character peak produced in PCI-MS complements an EI mass spectrum, and is integral in annotating an unknown mass peak. Up to date, breathomics study using GC-PCI-MS for peak annotation is still limited. Nonetheless, absence of a chromatographic separation in SIFT-MS complicates the unambiguous identification of target compounds, as shown in the cases of ethylphenol isomers, and propanol isomers (Table S-1). Isobaric discrimination sometimes become possible owing to the different relative abundance of the respective isotopologues, as exemplified by the case of phenol and dimethyl disulfide in the previous study^36^. As indicated in Table S-1, each compound exhibited varied MS profile individually in different platforms, depicting its difference in molecular structural elucidation. Additionally, some compounds cannot be detected in certain system, for instance branch chain alcohol and isomeric ethylphenol cannot be distinguished by SIFT-MS. Hence, data integration originating from different platforms (i.e. SIFT-MS and GC-MS) would improve the confidence of mass spectral annotation.

Unambiguous assignment of confirmed metabolites demands high standards for confidence as the result of matching retention time, isotopic ratio, and fragmentation pattern of their authentic standards^37^. High accuracy mass spectrometers such as orbitrap and time-of-flight MS can be complied in this strategy for disclosing the elemental composition of an unknown metabolite but caution must be taken as such instruments present challenge to precisely determine VOCs below 50 Dalton mass range due to extreme vacuum operation within the analyser. Derivatisation for ketones, acids and aldehydes prior to analysis has been commonly employed for targeted studies, thus improving the detectability of carbonyl functional VOCs via oximation or silylation^38–40^. In untargeted breathomics analysis, artifacts potentially introduced during the derivatization reaction would potentially distort the reliability of biomarker discovery. Other structural elucidation techniques such as spectroscopy and ultrasonography, in spite of providing the possibility to perform direct analysis, are incapable to serve the molecular identification purpose for compounds at the trace levels normally occurring in breath^41^.

Occurrence of oxygenated volatile compounds has been well related with oxidative stress and up-regulation of cell proliferation^42,43^, which were found in different biofluids such as serum, sputum and exhaled breath condensate^44,45^. Over-expression of oxygenated VOCs in body and breath was associated with cellular metabolic regulations such as glycolysis, apoptosis, loss of tumour suppressor genes and angiogenesis in the case of cancer^45,46^. Via simultaneous sampling of multiple TD tubes on exhaled breath from cancer patient, the proposed multi-platform strategy utilises different ionisation techniques, i.e. GC-EI-MS, GC-PCI-MS and SIFT-MS, to practically afford wealthy structural information on the VOCs present in the breath of cancer patients. Explicitly, elucidative details on the cancer VOCs shall provide extremely valuable information for critical cancer cellular studies.

## Conclusion

Multiple MS platforms namely GC-EI-MS, GC-PCI-MS and SIFT-MS were evaluated for compound annotation in VOCs and a orthogonal and combinatorial MS approach was applied in untargeted disease breathomics. When peak identification by GC-EI-MS analysis was uncertain, direct water infusion GC-PCI-MS with mild fragmentation provides complimentary molecular details for the polar VOC annotation. Molecular ion and fragments i.e. [M + H]^+^, followed by [M-17]^+^ and [M-73]^+^ observed in PCI-MS analysis could be potentially used as the diagnostic ions to distinguish the corresponding VOCs. SIFT-MS analysis offers unique specificity particularly on identification of aldehyde volatile according to the established kinetic database although isomeric VOCs are indistinguishable. Intercomparison between GC-MS and SIFT-MS responses displayed linear correlation at coefficient R^2^ over 0.92 while limit of detection for VOCs measurement using SIFT-MS ranged from 2 to 7 ppbV. Using the proposed multi-platform strategy, novel VOCs profile from oesophageal-gastric cancer breath could be reliably uncovered. Confirmation of the significant VOC identities would remarkably convenient for high-throughput quantitative targeted analysis. This has important implications for understanding of the oxygenated VOCs exhalation kinetics including for those with bifunctional grouping and influences strategies to establish robust validated model for non-invasive early cancer diagnosis.

## Electronic supplementary material

Supplementary Information
