# Supplementary material for: Cross-platform mass spectrometry annotation in breathomics of oesophageal-gastric cancer
Source: Sci Rep. 2018 Mar 23;8:5139. doi: 10.1038/s41598-018-22890-w (PMC5865157; doi:10.1038/s41598-018-22890-w)
Supplement: Supplementary file 1 — Supplementary Information [file 41598_2018_22890_MOESM1_ESM.pdf]

CROSS-PLATFORM MASS SPECTROMETRY ANNOTATION IN BREATHOMICS OF OESOPHAGEAL-GASTRIC CANCER

By

Sung-Tong Chin, Andrea Romano, Sophie LF Doran, George B. Hanna*

Division of Surgery, Department of Surgery and Cancer, Imperial College London, London W2 1NY, United Kingdom.

Supplementary Information

FIGURE S-1. Representative total ion chromatogram of breath VOC obtained using GC-EI-MS, GC-PCI-MS and mass spectrum of SIFT-MS of breath obtained in H3O+ scan mode.

TABLE S-1. Molecular details of VOC standards identified using GC, EI-MS, PCI-MS and SIFT-MS analysis. Volatile components were loaded onto TD tube using CSLR prior to MS analysis. All analysis was conducted on each different MS platforms in triplicate.

TABLE S-2. Demographic details of OG cancer patients recruited for breath analysis.

TABLE S-3. Database of SIFT-MS kinetic entries comprising number of reagent ions and product ions, and their kinetic coefficients correspondingly.

FIGURE S-1. Representative total ion chromatogram of breath VOC obtained using GC-EI-MS, GC-PCI-MS and mass spectrum of SIFT-MS of breath obtained in H_3O^+ scan mode.

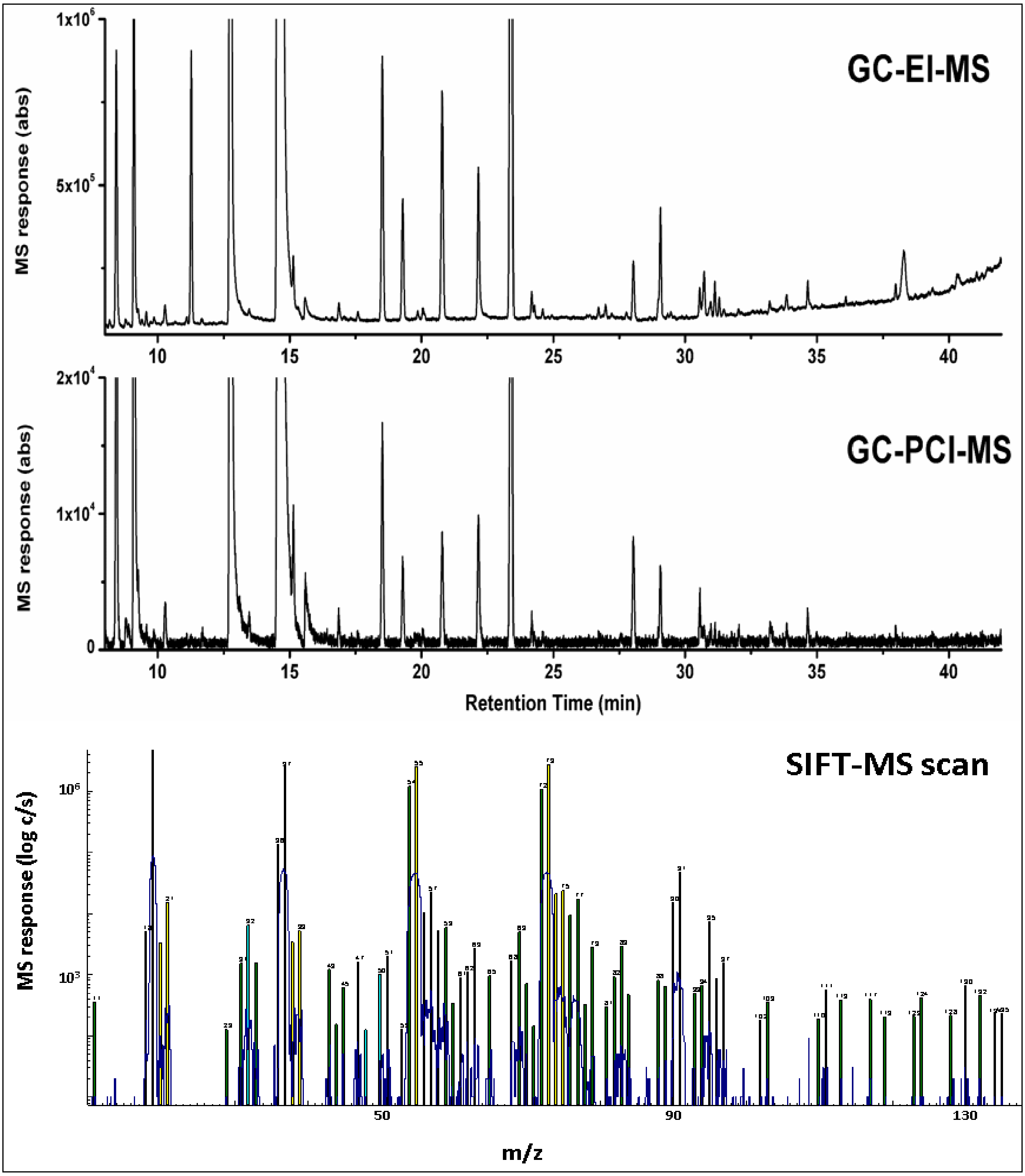

TABLE S-1. Molecular details of VOC standards identified using GC, EI-MS, PCI-MS and SIFT-MS analysis. Volatile components were loaded onto TD tube using CSLR prior to MS analysis. All analysis was conducted on each different MS platforms in triplicate.

Compound	CAS no.	RI $_{624}$	PCI-MS Mass spectrum	EI-MS Mass spectrum	SIFT-MS[§]	
			m/z (intensity)	m/z (intensity)	Precursors	reactants
**Phenols**						
phenol	108-95-2	1158	94(999)[a],95(809)[b]	94(999)[a],66(387),65(266),39(243)	H_3O^+	95,113
					NO^+	94,112
4-methylphenol; 3-methylphenol	106-44-5; 108-39-4	1241	107(999)[c],108(988)[a],109(697)[b]	108(999)[a],107(953),77(325),90(121)	H_3O^+	109,127
					NO^+	108,126
					O_2^+	108,126
2-ethylphenol	90-00-6	1286	123(999)[b],107(761),122(623)[a]	107(999),122(377)[a],77(287),79(158)	H_3O^+	123,141
					NO^+	122,140
					O_2^+	107,122,125,140
4-ethylphenol;	123-07-9;	1323	123(999)[b],107(761),122(623)[a]	107(999),122(377)[a],77(287),79(158)	H_3O^+	123,141
3-ethylphenol	620-17-7				NO^+	122,140
					O_2^+	107,122,125,140

**Ketones**						
acetone	67-64-1	498*	59(999)[b],58(363)[a]	43(999),58(487)[a]	H_3O^+	59,77
					NO^+	88
					O_2^+	43,58
2-butanone	78-93-3	608	73(999)[b],57(300)	43(999),72(250)[a],57(80)	NO^+	102
2-pentanone	107-87-9	716	87(999)[b],58(220),71(201)	43(999),86(197)[a],58(98),71(97)	NO^+	116
2-hexanone	591-78-6	850	101(999)[b],58(431),85(97)	43(999),58(664),85(119),71(84),100(180)[a]	NO^+	130
2-heptanone	110-43-0	976	115(999)[b],58(401)	43(999),58(549),71(133),114(60)[a]	NO^+	144
2-octanone	111-13-7	1089	129(999)[b],58(391)	43(999),58(859),71(189),128(50)[a]	NO^+	158
2-nonanone	821-55-6	1190	143(999)[b],58(403)	43(999),58(911),71(223),142(70)[a]	nd	nd
2-decanone	693-54-9	1281	157(999)[b],58(409)	58(999),43(845),71(377),156(40)[a]	H_3O^+	157,175
**Unsaturated Aldehydes**						
2-propenal	4170-30-3	479*	57(999)[b],55(400),56(314)	56(999)[a],27(954), 55(718)	H_3O^+	57,55,75,73
					NO^+	86,112
					O_2^+	28,55,56
2-butenal	123-73-9	683	71(999)[b],69(394),70(261)	41(999),39(899),70(736)[a],69(401)	H_3O^+	71,89
					NO^+	69
2-pentenal	1576-87-0	818	85(999)[b],84(366),55(172)	55(999),84(874) [a],41(508)	H_3O^+	85,103

					NO^+	83,114
2-hexenal	6728-26-3	947	99(999)[b],57(572),69(235), 83(326)	41(999),55(586),69(500),83(389), 98(160) [a]	H_3O^+	99,117
					NO^+	97,128
2-heptenal	18829-55-5	1068	113(999)[b],95(507),83(541), 57(593),68(158)	41(999),55(427),70(260),83(400)	H_3O^+	113,131
					NO^+	111,142
2-octenal	2548-87-0	1173	127(999)[b],109(811),57(579),	41(999), 55(845),70(706),83(617)	H_3O^+	127,145
			70(394),83(381)		NO^+	125,156
2-nonenal	18829-56-6	1268	141(999)[b],123(740),81(491), 57(477)	41(999),55(756),70(718),83(476), 96(220),122(120),140(40) [a]	H_3O^+	141,159
					NO^+	139,170
2,4-nonadienal	5910-87-2	1327	81(999)[d],139(950) [b],121(126)	81(999),41(201),67(164),138(77) [a]	nd	nd
2,4-decadienal	25152-84-5	1406	153(999) [b],81(908),135(151)	81(999),41(508),55(161),67(171), 152(60) [a]	H_3O^+	153,171
					NO^+	151
**Alcohols**						
1-propanol	71-23-8	576	59(999) [c],61(193) [b]	31(999),42(135),59(109) [c]	H_3O^+	43,61,79,97
					NO^+	59,77,95,113
2-propanol	67-63-0	506	59(999) [c],61(818) [b]	45(999),59(40) [c]	nd	nd
1-butanol	71-36-3	698	57(999)[d],56(454),73(85) [c]	56(999),31(981),41(876)	H_3O^+	57,75,93

2-methyl-1-propanol	78-83-1	654	57(999)[d],73(123)[c]	43(999),31(376),74(141)[a]	nd	nd
1-pentanol	71-41-0	829	71(999)[d],55(230),87(66)[c]	55(999),42(949),70(489),31(369)	H_3O^+	71,89,107,125
2-methyl-1-butanol	137-32-6	792	71(999)[d],57(306),56(272)	57(999),41(877),70(423),31(369)	nd	nd
**<u>Saturated Aldehydes</u>**						
propanal	123-38-6	489*	59(999)[b],58(512),57(315)	58(999)[a],29(880)	NO^+	55,57
butanal	123-72-8	596	73(999)[b],55(637),72(352)	44(999),43(786),72(734)[a],57(258)	NO^+	71
pentanal	110-62-3	724	69(999)[d],87(603)[b],58(318)	44(999),58(485),86(60)[a]	NO^+	85
hexanal	66-25-1	859	83(999)[d],56(360),101(196)[b]	44(999),56(819),43(550), 72(160),82(120)	NO^+	99
heptanal	111-71-7	986	97(999)[d],70(384),55(234), 115(135)[b]	70(999),44(883),43(836), 55(779),81(320), 96(180)	NO^+	113
benzaldehyde	100-52-7	1087	107(999)[b],105(494),106(480)	106(999)[a],105(950),77(889),51(495)	nd	nd
octanal	124-13-0	1099	111(999)[d],69(963),84(517), 129(239)[b]	43(999),44(808),56(657),84(550)	NO^+	127
nonanal	124-19-6	1200	69(999),57(588),83(576),98(339), 125(248)[d],143(208)[b]	57(999),41(893),43(879),44(715), 70(421), 98(406), 82(300)	NO^+	141
decanal	112-31-2	1291	83(999),57(554),97(461),69(363), 112(193),157(185)[b]	43(999),41(801),57(621),44(539), 70(471), 82(360), 112(200)	NO^+	155
undecanal	112-44-7	1375	61(999),97(941),83(766),71(597),	41(999),43(902),57(699),82(461),	nd	nd

			111(320),171(209) [b]	44(406),67(346), 126(100)		
**Acids**						
acetic acid	64-19-7	674	61(999) [b],60(210)	43(999),45(903),60(747) [a]	H_3O^+	61,79,97
					NO^+	90
propanoic acid	79-09-4	790	75(999) [b],74(459),57(259)	74(999) [a],45(900),57(466)	H_3O^+	73,75,93,111
					NO^+	104,122,57,55
butanoic acid	107-92-6	900	89(999) [b],60(480),71(239),73(145)	60(999),73(324),41(162)	H_3O^+	89,107,125
					NO^+	118,136,71
pentanoic acid	109-52-4	1012	103(999)[b],60(423),85(253),73(194)	60(999),73(356),41(178)	H_3O^+	103,121,139
hexanoic acid	142-62-1	1112	117(999)[b],60(322),99(266),73(187)	60(999),73(445),87(134),41(196)	H_3O^+	117,135,153

RI_{624} indicates retention index on ZB-624 column; [a] indicates M^+; [b] indicates $[M+H]^+$; [c] indicates $[M-H]^+$; [d] indicates $[M-17]^+$;

[§]Information for rate constant and coefficient of each percursor and reactant was listed in supplementary information;

nd indicates not detected; * denotes estimated retention index from extrapolation.

TABLE S-2. Demographic details of OG cancer patients recruited for breath analysis.

	Patient ID																				
**Factor**	**1**	**2**	**3**	**4**	**5**	**6**	**7**	**8**	**9**	**10**	**11**	**12**	**13**	**14**	**15**	**16**	**17**	**18**	**19**	**20**	**21**
Age	56	71	61	78	74	44	79	43	77	61	67	61	60	66	63	56	52	65	52	48	69
Gender	M	M	M	M	M	M	M	M	F	M	F	M	M	M	M	M	M	M	M	M	M
Race	C	C	C	C	C	AR	C	C	C	C	C	C	AS	C	AS	C	AR	C	C	C	C
Smoking history (pack year)	10	10	0	30	0	20	75	0	30	20	0	20	0	10	10	15	10	5	10	10	5
Alcohol history (unit week)	10	12	30	2	8	0	5	0	5	1	0	1	3	4	0	5	0	5	16	5	10
ASA grade	3	2	2	2	3	1	3	2	2	2	2	2	1	2	2	2	1	2	1	1	2
Diabetes	N	N	N	N	Y	N	N	N	N	Y	Y	Y	N	N	N	N	N	N	N	N	N
Pulmonary impairment	N	N	N	N	N	N	Y	N	N	N	N	N	N	N	N	N	N	N	N	N	N
Ischaemic heart disease	N	N	N	N	Y	N	Y	N	N	N	N	N	N	N	N	N	N	N	N	N	Y
Hypertension	Y	Y	Y	N	Y	N	N	N	N	Y	N	Y	Y	N	N	N	N	N	Y	Y	N
Asthma	Y	N	N	N	N	N	N	N	N	N	N	N	N	N	N	N	N	N	Y	N	N

M: Male, F: Female, C: Caucasian, AR: Arabian, AS: Asian.

TABLE S-3. Database of SIFT-MS kinetic entries comprising number of reagent ions and product ions, and their kinetic coefficients correspondingly.

2,4-decadienal(H3O+)

4 precursors /rate constant: 19 /4.9e-9 1.0, 37 /3.0e-9 1.0, 55 /2.7e-9 1.0, 73 /2.6e-9 1.0

2 products /rate constant: 153 /1, 171 /1,

2,4-decadienal(NO+)

3 precursors /rate constant: 30 /4.2e-9 1.0, 48 /1e-9 1.0, 66 /1e-9 1.0

1 product /rate constant: 151 /1.2

butanal(NO+)

3 precursors /rate constant: 30 /3.3e-9 1.0, 48 /1e-9 1.0, 66 /1e-9 1.0

1 products /rate constant: 71 /1

pentanal(NO+)

3 precursors/rate constant: 30 /3.0e-9 1.0, 48 /1e-9 1.0, 66 /1e-9 1.0

1 product /rate constant: 85 /1.2

hexanal(NO+)

3 precursors /rate constant: 30 /3.0e-9 1.0, 48 /1e-9 1.0, 66 /1e-9 1.0

1 product /rate constant: 99 /1

heptanal(NO+)

3 precursors/rate constant: 30 /3.3e-9 1.0, 48 /1e-9 1.0, 66 /1e-9 1.0

1 product /rate constant: 113 /1

octanal(NO+)

3 precursors /rate constant: 30 /3.0e-9 1.0, 48 /1e-9 1.0, 66 /1e-9 1.0

1 product /rate constant: 127 /1

nonanal(NO+)

3 precursors /rate constant: 30 /3.3e-9 1.0, 48 /1e-9 1.0, 66 /1e-9 1.0

1 product /rate constant: 141 /1

decanal(NO+)

3 precursors /rate constant: 30 /3.3e-9 1.0, 48 /1e-9 1.0, 66 /1e-9 1.0

1 product /rate constant: 155 /1

acrolein(H3O+)

4 precursors /rate constant: 19 /4.2e-9 1.0, 37 /3.3e-9 1.0, 55 /3.0e-9 1.0, 73 2.8e-9 1.0

1 product /rate constant: 39 /1

acrolein(H3O+)

4 precursors /rate constant: 19 /4.2e-9 1.0, 37 /3.3e-9 1.0, 55 /3.0e-9 1.0, 73 /2.8e-9 1.0

4 products /rate constant: 57 /1, 55 /-0.006, 75 /1, 73 /-0.008

acrolein(NO+)

3 precursors /rate constant: 30 /2.1e-9 1.0, 48 /1.6e-9 1.0, 66 /1.5e-9 1.0

2 products /rate constant: 86 /1, 112 /1

acrolein(O2+)

1 precursors /rate constant: 32 /4.9e-9 1.0

3 products /rate constant: 28 /1, 55 /1, 56 /1

acetaldehyde(H3O+)

4 precursors /rate constant: 19 /3.7e-9 1.0, 37 /3.0e-9 1.0, 55 /2.7e-9 1.0, 73 /2.6e-9 1.0

4 products /rate constant: 45 /1.0, 63 /1.0, 81 /1.0, 99 1.0

acetaldehyde(NO+)

1 precursor /rate constant: 30 /0.6e-9 1.0

1 product /rate constant: 43 /1.0

acetone(H3O+)

4 precursors /rate constant: 19 /3.9e-9 1.0, 37 /3.3e-9 1.0, 55 /2.5e-9 1.0, 73 /2.4e-9 1.0

2 products /rate constant: 59 /1.0, 77 /1.0

acetone(NO+)

1 precursor /rate constant: 30 /2.2e-9 1.0

1 product /rate constant: 88 /1.0

acetone(O2+)

1 precursor /rate constant: 32 /3.1e-9 1.0

2 products /rate constant: 43 /1.0, 58 /1.0

ethanol(H3O+)

4 precursors /rate constant: 19 /2.7e-9 1.0, 37 /2.3e-9 1.0, 55 /2.1e-9 1.0, 73 /1e-11 1.0

3 products /rate constant: 47 /1.0, 65 /1.0, 83 /1.0

methanol(H3O+)

4 precursors /rate constant: 19 /2.4e-9 1.0, 37 /1.9e-9 1.0, 55 /1.9e-9 1.0, 73 /1e-11 1.0

3 products /rate constant: 33 /1.0, 51 /1.0, 69 1.0

propanol(H3O+)

4 precursors /rate constant: 19 /2.7e-9 1.0, 37 /2.3e-9 -0.1, 55 /2.2e-9 -0.1, 73 /2.1e-9 -0.1

1 product /rate constant: 43 /1.0, 61 /1.0, 79 /1.0, 97 /1.0

propanol(NO+)

3 precursors /rate constant: 30 /2.4e-9 1.0, 48 /2.0e-9 1.0, 66 /1.9e-9 1.0,

4 products /rate constant: 59 /1.0, 77 /1.0, 95 /1.0, 113 /1.0

butanol(H3O+)

4 precursors /rate constant: 19 /2.8e-9 1.0, 37 /2.2e-9 1.0, 55 /1.9e-9 1.0, 73 /1.8e-9 1.0

5 products /rate constant: 57 /1.0, 55 /-0.006, 75 /1.0, 73 /-0.008, 93 /1.0

pentanol(H3O+)

4 precursors /rate constant: 19 /2.8e-9 1.0, 37 /2.2e-9 1.0, 55 /1.9e-9 1.0, 73 /1.8e-9 1.0

4 products /rate constant: 71 /1.0, 89 /1.0, 107 /1.0, 125 /1.0

phenol(H3O+)

4 precursors /rate constant: 19 /2.4e-9 1.0, 37 /1.9e-9 1.0, 55 /1.9e-9 1.0, 73 /5e-10 1.0

2 products /rate constant: 95 /1.0, 113 /1.0

phenol(NO+)

3 precursors /rate constant: 30 /2.2e-9 1.0, 48 /1.6e-9 1.0, 66 /1.3e-9 1.0

2 products /rate constant: 94 /1.0, 112 /1.0

methyl_phenol(H3O+)

4 precursors /rate constant: 19 /2.8e-9 1.0, 37 /1.7e-9 1.0, 55 /1.2e-9 1.0, 73 /5e-10 1.0

2 products /rate constant: 109 /1.0, 127 /1.0

methyl_phenol(NO+)

3 precursors /rate constant: 30 /2.2e-9 1.0, 48 /1.6e-9 1.0, 66 /1.3e-9 1.0

2 products /rate constant: 108 /1.0, 126 /1.0,

methyl_phenol(O2+)

1 precursor /rate constant: 32 /2.2e-9 1.0

2 products /rate constant: 108 /1.0, 126 /1.0

ethyl_phenol(H3O+)

4 precursors /rate constant: 19 /2.8e-9 1.0, 37 /1.7e-9 1.0, 55 /1.2e-9 1.0, 73 /5e-10 1.0

2 products /rate constant: 123 /1.0, 141 /1.0

ethyl_phenol(NO+)

3 precursors /rate constant: 30 /2.3e-9 1.0, 48 /1.6e-9 1.0, 66 /1.3e-9 1.0

2 products /rate constant: 122 /1.0, 140 /1.0

ethyl_phenol(O2+)

1 precursor /rate constant: 32 /2.2e-9 1.0

4 products /rate constant: 107 /1.0, 122 /1.0, 125 /1.0, 140 /1.0

acetic_acid(H3O+)

4 precursors /rate constant: 19 /2.6e-9 1.0, 37 /1.9e-9 1.0, 55 /1.9e-9 1.0, 73 ,1.8e-9 1.0

3 products /rate constant: 61 /1.0, 79 /1.0, 97 /1.0

acetic_acid(NO+)

3 precursors /rate constant: 30 /1.5e-9 1.0, 48 /5e-10 1.0, 66 /5e-10 1.0

1 product /rate constant: 90 /1.0

propanoic_acid(H3O+)

4 precursors /rate constant: 19 /2.7e-9 1.0, 37 /1.9e-9 1.0, 55 /1.9e-9 1.0, 73 /1.9e-9 1.0

4 products /rate constant: 73 /-0.008, 75 /1.0, 93 /1.0, 111 /1.0

butyric_acid(H3O+)

4 precursors /rate constant: 19 /2.9e-9 1.0, 37 /1.9e-9 1.0, 55 /1.9e-9 1.0, 73 /1.9e-9 1.0

3 products /rate constant: 89 /1.0, 107 /1.0, 125 /1.0

pentanoic_acid(H3O+)

4 precursors /rate constant: 19 /2.4e-9 1.0, 37 /1.9e-9 1.0, 55 /1.9e-9 1.0, 73 /1.7e-9 1.0

3 products /rate constant: 103 /1.0, 121 /1.0, 139 /1.0

hexanoic_acid(H3O+)

4 precursors /rate constant: 19 /2.4e-9 1.0, 37 /1.9e-9 1.0, 55 /1.9e-9 1.0, 73 /1.7e-9 1.0

3 products /rate constant: 117 /1.0, 135 /1.0, 153 /1.0

propionic_acid(NO+)

3 precursors /rate constant: 30 /1.5e-9 1.0, 48 /5e-10 1.0, 66 /5e-10 1.0

4 products /rate constant: 104 /1.0, 122 /1.0, 57 /1.0, 55 /-0.006

butyric_acid(NO+)

3 precursors /rate constant: 30 /1.9e-9 1.0, 48 /7e-10 1.0, 66 /7e-10 1.0

3 products /rate constant: 118 /1.0, 136 /1.0, 71 /1.0

formaldehyde

1 precursor /rate constant: 19 /2.7e-9 1.0

1 product /rate constant: 31 /1.0

noise(H3O+)

1 precursor /rate constant: 19 /3.1e-9 1.0

3 products /rate constant: 10 /1.0, 24 /1.0, 150 /1.0

butanone(NO+)

1 precursors /rate constant: 30 /2.5e-9 1.0

1 product /rate constant: 102 /1.0

pentanone(NO+)

1 precursors /rate constant: 30 /2.5e-9 1.0,

1 product /rate constant: 116 /1.0

hexanone(NO+)

1 precursors /rate constant: 30 /2.5e-9 1.0

1 product /rate constant: 130 /1.0

heptanone(NO+)

1 precursors /rate constant: 30 /2.5e-9 1.0

1 product /rate constant: 144 /1.0

octanone(NO+)

1 precursors /rate constant: 30 /2.5e-9 1.0

1 product /rate constant: 158 /1.0
